# A comparative cradle-to-gate life cycle assessment of three cotton stalk waste sustainable applications

**DOI:** 10.1038/s41598-023-47817-y

**Published:** 2023-11-27

**Authors:** Rana Adel Ibrahim, Hatice Inan, Irene S. Fahim

**Affiliations:** 1 Smart Engineering Systems Research Center, Nile University, Giza, Egypt; 2 School of Industrial Engineering, Nile University, Giza, Egypt

**Keywords:** Climate sciences, Environmental sciences

## Abstract

This paper presents a novel approach to utilizing agricultural waste. It compares three different applications for cotton stalks: fabrication of wood composites, bioethanol production, and biogas cradle-to-gate Life cycle assessment production processes. Cotton cultivation generates a lot of debris, mostly cotton stalks, which are incinerated or landfilled, Sustainable resource management is critical for maintaining the ecosystem, and economic stability, and promoting social fairness since it ensures the long-term availability of resources while minimizing environmental damage. The investigation uses the Ecological Footprint, Impact 2002 +, Global Warming Damage Potential, Greenhouse Gas Protocol, Recipe Midpoint, Ecosystem Damage Potential, and CML IA Baseline—open LCA-enabled environmental sustainability assessments. The analysis showed that bioethanol has a lower carbon footprint and climate change impact than both wood composite and biogas production processes, as a result, this could cause a preference for bioethanol production as an environmentally friendly strategy for cotton stalks utilization. While human toxicity was higher in the biogas production process, it emits less fossil CO_2_ than biogenic CO_2_. The total climate change of wood composite, bioethanol, and biogas production processes was 0.01761, 0.011300, and 0.01083 points, respectively. This research helps accomplish wider ecological and economic aims by giving insights into sustainable waste management practices.

## Introduction

It is essential to change waste management and resource usage practices to achieve environmental sustainability and long-term economic viability. The issue of waste, particularly within the agricultural sector, is a pressing one that necessitates attention within the framework of sustainable resource utilization. Cotton is cultivated on a global scale, spanning an extensive land area of 32.6 million hectares, and including the participation of around 111 countries. The cultivation process generates considerable cotton waste, predominantly in the form of cotton stalks^1^. Cotton farming generates a significant amount of waste, ranging from 1 to 2 tons per hectare, following the completion of the harvesting process^2,3^. Currently, the estimated annual global availability of cotton stalks ranges from 90.3 to 129 million tonnes^4^. It is anticipated that this availability will increase in the future.

The recycling of agricultural waste is vital for promoting environmental sustainability. Cotton stalk waste serves as a prime example in this context. Combusting these byproducts releases significant amounts of carbon dioxide and other greenhouse gases, exacerbating climate change. Moreover, this traditional disposal method results in the loss of valuable biochemical compounds found in cotton stalks^5^. Cotton stalks are a potential source of raw materials to produce bioethanol, biogas, and composite building materials due to their high cellulose (33–36%), hemicellulose (9–18%), and lignin (25–31%) content^6–8^. Their biochemical composition promotes the production of biogas through anaerobic digestion as well as supports the conversion into fermentable sugars for bioethanol both of which have the potential to be used as renewable and environmentally benign energy sources. This aligns with overarching objectives related to the environment and sustainability, as highlighted by Acevedo et al.^9^ and Ding et al.^5^. Cotton stalk waste in construction is a novel solution to deforestation and sustainability^10^. With 11.9 gigatons of emissions, the construction industry accounts for 37% of energy-related greenhouse gas emissions^5^. Wood and wood products are essential in construction. However, rising urbanization and limited energy resources are moving civilizations toward wood-based energy and construction materials, increasing wood consumption and deforestation^11^. This desire for sustainable wood products has led to unregulated forest usage and wood scarcity, driving research into alternate construction materials^12^. The wood products industry's resource restrictions have prioritized research in this field^13^.

Life Cycle Assessment (LCA) and Life Cycle Impact Assessment (LCIA) are important methodologies used to evaluate the environmental impact of alternative resources^14,15^. There is an urgent requirement to enhance the efficiency of biomass conversion processes for cotton stalks in the energy and construction industries. This optimization should reduce harmful environmental effects while upholding sustainability ideals. Activities can be evaluated through several life cycle stages, such as "cradle to cradle" or "cradle to gate," depending on their intended use. These analyses provide thorough insights into environmental implications, assisting decision-makers and stakeholders in making more informed decisions that are sustainable^14^.

There is a limited amount of available literature on Life Cycle Assessment (LCA) pertaining to the potential utilization of cotton stalks for energy generation and their application as construction materials. The current body of research encompasses individual Life Cycle Assessment (LCA) investigations pertaining to the production of bioethanol^16–18^, biogas^19,20^, and construction materials^21,22^. However, a comprehensive study that integrates and contrasts the applications of these substances in these respective domains is lacking. This phenomenon results in a notable deficit in information for individuals responsible for making decisions, posing challenges to pursuing sustainable development and safeguarding the environment. Within this context, the necessity for a life cycle assessment (LCA) investigation that thoroughly evaluates the sustainability and environmental ramifications of cotton stalks suggests that this study will address a significant void in the existing body of scholarly work. The wood products industry is fully dependent on natural wood resources as a raw material input. Because of expanding populations and economic development, more wood has been cut than replanted in some locations as worldwide demand for wood products has expanded over time. In some places of the world, unsustainable timber harvesting practices and deforestation have decreased natural forest stock. Furthermore, climate change is aggravating pest outbreaks, wildfires, and droughts, that harm commercial forestry. With natural forests unable to recover quickly enough to restore supply, the wood products industry today faces a scarcity of raw wood required for manufacturing, resulting in a raw material shortage^23^.

The objective of this study is to employ Life Cycle Assessment (LCA) and Life Cycle Impact Assessment (LCIA) approaches to assess the environmental and economic implications associated with the utilization of cotton stalks in energy generation and as construction materials. The primary objective is to offer significant perspectives for individuals and organizations involved in the building and energy industries, with the aim of encouraging a transition towards sustainable methodologies. Through the examination of the existing knowledge deficit pertaining to the sustainability of cotton stalk waste within various industries, the outcomes of this study will provide valuable guidance for stakeholders and contribute to the advancement of environmental and sustainable development endeavors. The analysis utilizes a range of environmental sustainability indicators that are compatible with the Open LCA software^24^. These metrics include the Ecological Footprint, Impact 2002 +, Global Warming Damage Potential, Greenhouse Gas Protocol, Recipe Midpoint, Ecosystem Damage Potential, and CML IA Baseline.

## Methodology

A comparative life cycle assessment has been performed using cotton stalk waste in three fruitful environmental applications. The life cycle assessment had four stages which are, scope description, lifecycle inventory, lifecycle impact analysis, and lifecycle impact interpretation. Open-LCA software has been used, Open-LCA is an open-source and free software. This promoted LCA community participation, openness, and development. Open LCA had a user-friendly interface that made it easier to create and manage LCA models. It offered simple tools for the entry of information and parameterization making it suitable for beginners as well as experts. Also, it offered a wide range of capabilities for conducting LCA studies, such as inventory evaluation, impact assessment, sensitivity evaluation, and documentation. It has supported a variety of impact assessment approaches and provided a lot of flexibility when setting system boundaries and modeling product life cycles^25^. Costs and social aspects could also be modeled. Datasets could be readily imported and exported and can be shared online. And it could produce ecological product declarations^26^. The Agribalyse database is a comprehensive database that includes a wide range of agricultural and food-related processes. It provided extensive lifecycle inventory (LCI) statistics for agricultural goods such as crop agriculture, animal breeding, and processing of food. This wide range of information allowed for detailed and reliable evaluations of agricultural systems' environmental implications. Transparent and documented data Sources: Agribalyse promoted transparency and dependability by recording and referencing the data providers for each step. This allowed users to verify and validate the data, confirming the LCA results' authenticity^27^. The Ecoinvent 3.6 database included industrial sectors such as energy production, manufacturing, agriculture, transportation, and waste management. It contained information on thousands of different processes and materials, allowing for a thorough examination of environmental implications across industries and product life cycles. High data quality: The EcoInvent database is well known for its stringent data collecting and quality control processes. To ensure accuracy and reliability, the data was subjected to comprehensive peer review and verification. Because of the high data quality, LCA studies employing Ecoinvent data are trustworthy and robust^28^. This research was an attempt to examine the ecological impacts of three different cotton stalk applications. The LCIA's scope for cotton stalk uses involved examining the impact on the environment of using cotton stalks for bioethanol production, biogas, and wood composite, for example. The study's goal would be to measure and assess the environmental impacts associated with each use, such as greenhouse gas emissions, land use, water consumption, and potential implications on biodiversity. The research provided vital insights into the environmental consequences of the three cotton stalk applications by completing a full LCIA, which could serve to inform decision-making processes and identify potential areas for improvement in terms of sustainability and resource management. The results also offered substitute material for the one with the greatest environmental impact. The life cycle assessment was intended to quantify the environmental impacts of cotton stalks' sustainable applications. The life cycle comparative study provided suggestions for waste recycling and utilization in an appropriate manner and provided sustainable solutions for enhancing environmental behavior. The impact results of this study were expected to be useful in both the construction and energy sectors. Also, it could be helpful in the agricultural sector for the farmers to perform both economically and environmentally friendly practices. There were three approaches utilizing cotton stalk waste for the life cycle impact assessment conducted, Cradle- to gate lifecycle assessment was performed to determine the carbon impact of a product beginning with the extraction of raw materials (cradle), continuing through the production process, and culminating at the factory gate when the final product is ready for delivery. Any other activities that were common to all scenarios, such as those between the factory gate and disposal, were excluded from the analysis.

### Key parameters and indicators in LCA

- Greenhouse gases (GHG) emissions: including carbon dioxide (CO_2_), methane (CH4), and nitrous oxide (N_2_O), are crucial in determining the impact of climate change. LCA enabled the measurement and quantification of these emissions at various phases of a product's life cycle. LCA provided an in-depth assessment of the carbon footprint and global warming potential of the system under investigation by quantifying these emissions in carbon dioxide equivalents (CO_2_e), which compensated for their varying global warming potentials. This could allow decision-makers to comprehend and reduce the effects of climate change on the evaluated system, supporting the creation of measures to minimize GHG emissions and, promoting environmental sustainability.
- Ecotoxicity: Ecotoxicity indicators had significance in LCA because they assessed the possible hazardous effects of chemicals on ecosystems and organisms. The effects of pollutants and waste discharges on aquatic and terrestrial ecosystems were evaluated using these indicators. Ecotoxicity markers provided useful insights into the possible ecological disturbance caused by the examined system by considering factors such as substance persistence, bioaccumulation, and toxicity. They aided in the identification of areas for improvement and assisted decision-making to reduce the environmental impact and promote sustainable practices. LCA contributed to the protection and preservation of ecosystems and the creatures that have depended on them by assessing environmental toxicity.
- Energy Consumption: Energy consumption is an important factor in determining the environmental impact of a product's life cycle. It calculated the amount of energy used at several stages, such as raw material extraction, manufacturing, transportation, product usage, and end-of-life operations. By evaluating energy usage, useful insights into the system's energy efficiency and its environmental effects were achieved. The measurement could be stated in joules or kilowatt-hours, allowing for comparisons and evaluations across various items or processes. Understanding energy consumption patterns over the life cycle allows for more informed decision-making and the execution of solutions to improve energy efficiency and reduce environmental impact.
- Human Health implications: LCA included indicators to examine the potential consequences on human health at different phases of a product's life cycle. Human toxicity and carcinogenicity were assessed in the context of hazardous substance exposure. LCA assisted in identifying possible dangers and selecting solutions to reduce health hazards by assessing human health implications. This data-informed decision-making in order to support the development of safer products and procedures. LCA contributed to the preservation and improvement of human health by taking into account the health consequences connected with a product's life cycle, encouraging the implementation of environmentally friendly and safer alternatives in a variety of sectors.
- Resource Depletion: LCA considered the depletion of finite resources such as fossil fuels, minerals, and water. For the estimation of the consequences of resources that are not renewable, indicators such as fossil fuel depletion potential and mineral extraction potential were utilized. LCA provided insights into the long-term sustainability of the system being studied by analyzing resource depletion. These markers aided in the identification of the ecological repercussions of resource extraction and use, allowing for the creation of resource-efficient alternatives. Decision-makers could make well-informed decisions to reduce reliance on finite resources and promote the use of more sustainable and renewable alternatives by addressing the depletion of resources in LCA.
- Water Demand and Pollutants: Water use and pollution are critical issues in LCA. Water consumption evaluation entailed estimating the amount of water used at each stage of the product's life cycle, which included the extraction process, production, product use, and end-of-life activities. Furthermore, LCA assessed the potential implications on the availability of water, such as water scarcity or contamination. Assessing the use of water offers useful insights into the water footprint and potential ecological effects of the evaluated system. This data assisted decision-making by exposing areas where water utilization can be improved, supporting sustainable water management practices, and minimizing the adverse effects on water resources.
- IMPACT 2002 + life cycle impact assessment methodology provided a workable application of a combined midpoint/damage approach, which connected all sorts of life cycle inventory results (elementary flows and other interventions). New concepts and approaches, particularly for the comparative assessment of human toxicity and ecotoxicity, have been created for IMPACT 2002 +. IMPACT 2002 + the technique gave description parameters for about 1500 different life cycle inventories^29^.

### Allocation methods and system boundaries

Allocation methods and limits of the system are essential parts of LCA. They defined the boundaries and scope of the study, as well as the distribution of environmental burdens across numerous activities or products over the course of a life cycle. System expansion was the allocation method that considered the effects of alternative processes or technology. The allocation technique chosen was determined by the unique aims and context of the LCA research and should be explicitly justified^30^.

### Scope description and assumptions

The initial phase of this research involved detailing the scope and underpinning assumptions of the study. The research goal was to verify the primary production parameters and significant variances in GHG emission through a comprehensive comparison of the life cycle of bioethanol, biogas, and wood composite production and proposed practical strategies for sustainable waste utilization. Assessing the environmental impact of the three applications of cotton stalk waste, namely: wood composite production, ethanol production, and biogas production. This evaluation adopted a cradle-to-gate approach, encompassing every process from cotton stalk waste generation to the production of the aforementioned applications. One critical assumption in this study pertained to the transportation of cotton stalk waste, which was included within the scope. The specific production processes for wood composites, bioethanol, and biogas were inferred based on existing literature, under the assumption that these processes were replicable with cotton stalk waste as the input. For instance, the production of the wood composite was employing soybean adhesive^31^. The experimental work of the wood composite production process was performed at Nile University, Egypt. The study’s cotton stalk mass assumption was that 1000 kg of cotton stalk waste was utilized across the three applications. This standardization facilitated a balanced comparison of the environmental impacts associated with each application. The assumptions and scope defined at this stage guided the subsequent phases of the lifecycle inventory, lifecycle impact analysis, and lifecycle impact interpretation.

### Life-cycle inventory

For the life cycle inventory stage of the study, Open LCA software was employed to generate the requisite data. Specifically, data from eco-invent v3.6 and the Agribalyse databases were imported into the software to ensure a comprehensive account of environmental inputs and outputs. After defining the process variables necessary for the three uses of cotton stalk waste, the software was used to calculate the necessary quantities of materials involved in each process. The inventory data that made up the life cycle inventory (LCI) for the chosen scenarios covered in this study are mostly data on raw materials and energy consumed obtained from prior studies, onsite data, and ecoinvent 3.6 databases. Based on the management methods previously described, the LCI is discussed. Both the input allocation procedure and the output allocation process used the product's mass. This step allowed for a detailed account of resource usage and waste generation across the lifecycle stages of each cotton stalk waste application, forming the basis for the subsequent lifecycle impact analysis.

### LCA impact assessment methods

The Life Cycle Impact Assessment (LCIA) part of this project made use of twelve different techniques drawn from the Ecoinvent database. These techniques, which included ecological footprint, IMPACT 2002 +, IMPACTWORLD +, ecosystem damage potential, and recipe midpoint, have been chosen to ensure a thorough understanding of the environmental effects related to the applications for cotton stalk waste that have been aforementioned. Along with these techniques, the study evaluated many impact categories. These included consequences on human health, terrestrial ecotoxicity, total CO_2_ emissions, land usage, and human habitation.

### Results interpretation

The final stage of this inquiry involved the examination of the results obtained from the LCIA. This phase integrated the LCIA outcomes and comprehensively analyzed the ecological impacts of every utilization of cotton stalk residue. This involved a systematic examination of the deductions and ramifications, furnishing insight into the ecological impacts associated with every waste utilization. The analysis of these results provided valuable insight that facilitated informed decision-making regarding the sustainable utilization and management of cotton stalk waste.

### Impact categories

Most studies that examined the environmental impact of the biogas system focused mainly on impacts, such as acidification, eutrophication, eco-toxicity, ozone depletion, fossil depletion, and climate change. Land use is an essential impact category, especially in biogas systems based on biomass resources, which may have an impact on biodiversity and habitat conservation. Several researchers used the lifecycle impact assessment approach, Eco-indicator 99, to assess the three damage categories (endpoint impacts): human health, resource depletion, and ecosystem quality. Differentiation criteria were employed to translate and integrate lifecycle inventory (LCI) data into representative indications of impact on three areas of protection. However, the endpoint method had substantial uncertainties, since unmodeled impacts would not be considered. Endpoints were at the end of the cause-effect chain and were used to aggregate the effects of stressors with distinct modes of action. Considering the cause-effect chain up to the endpoint stage would make the indicator more ecologically relevant but would result in large uncertainty because of a lack of adequate models and data. This uncertainty could have led to incorrect conclusions and limited the ability to compare different studies. Some studies mainly focused on greenhouse gases. The amount of greenhouse gas emissions, global warming potential (GWP), and greenhouse gas management potential (GMP) were all analyzed. Cumulative energy demand (CED) is the four types of demand for energy. These indicators were estimated in several studies to assess the energy performance of biogas production. Several studies on biogas production have found that the energy produced from biogas produces less greenhouse gas emissions than fossil fuels^29^ (Table 1).

**Table 1:** Inventory data for the three scenarios.

	Bioethanol	Biogas	Wood composite
Inputs (kg/hr)	1000 kg/hr Cotton stalks 1000 L/hr Water 100 kg/hr Enzyme 100 kg/hr NaOH,50%in H2Oa, 100 kg/hr fermentation activator 2.27–3.35 MJ/L EtOH Energy consumption for the ethanol production process^32^	1000 kg/hr Cotton stalks 100 L/hr Water 100 kg/hr Inoculum 100 kg/hr NaOH, 50%in H2Oa, 186 kWel/ton Energy consumption for the biogas production process^33^	1000 kg/hr Cotton stalks 100 L/hr Water 300 kg/hr soybean adhesive 100 kg/hr formaldehyde 2000–3000kWh/ton Energy consumption for chipping with hammer milling 10 MJ energy consumption for hot pressing^34^
Outputs	246.67 L/kg Bioethanol^32^ From 232 to 672 L/L EtOH Wastewater^35^ 52.5 MJ/ L EtOH Thermal energy costs^32^	192 L/kg Biogas^36^ 25 L/hr Waste water	160 m^3^/ton Wood composite 25/L wastewater
Emissions			
CO_2_	8.475 kg/L	2.586 kg/L	19.38 kg/m^3^
CO	3.002 kg/L	7.135 kg/L	6.90 kg/m^3^
NOX	4.863 kg/L	1.932 kg/L	3.85 kg/m^3^
SO_2_,	2.494 kg/L	0.065 kg/L	0.186 kg/m^3^
H_2_SO_4_	3.42 kg/L	0.047 kg/L	3.45 kg/m^3^
CH_4_	1.267 kg/L	5.67 kg/L	1.532 kg/m^3^

### Ethanol production scenario

**Figure 1:**
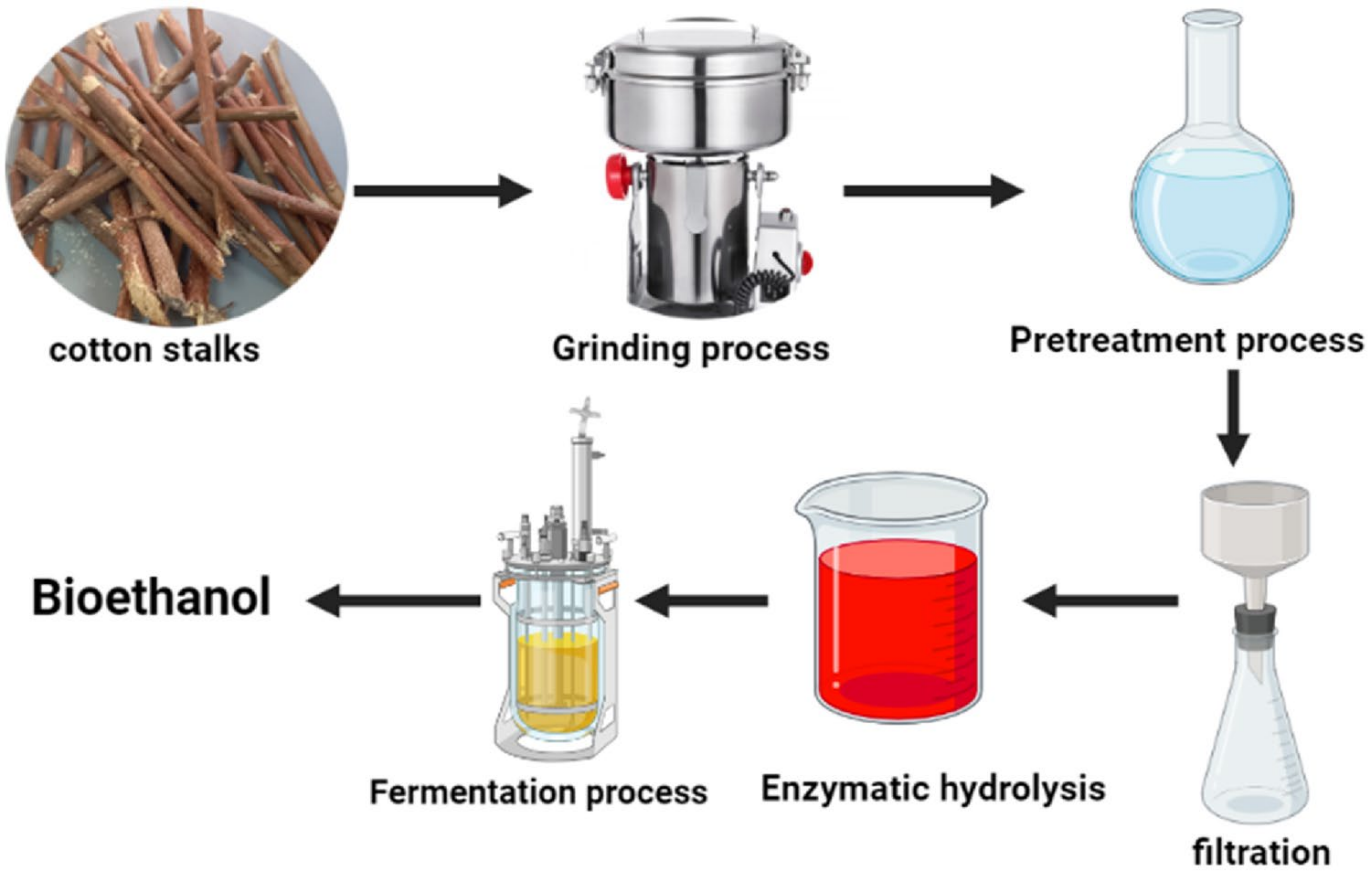
Diagram of ethanol production process flow showing the steps for lignocellulosic ethanol production.

**Figure 2:**
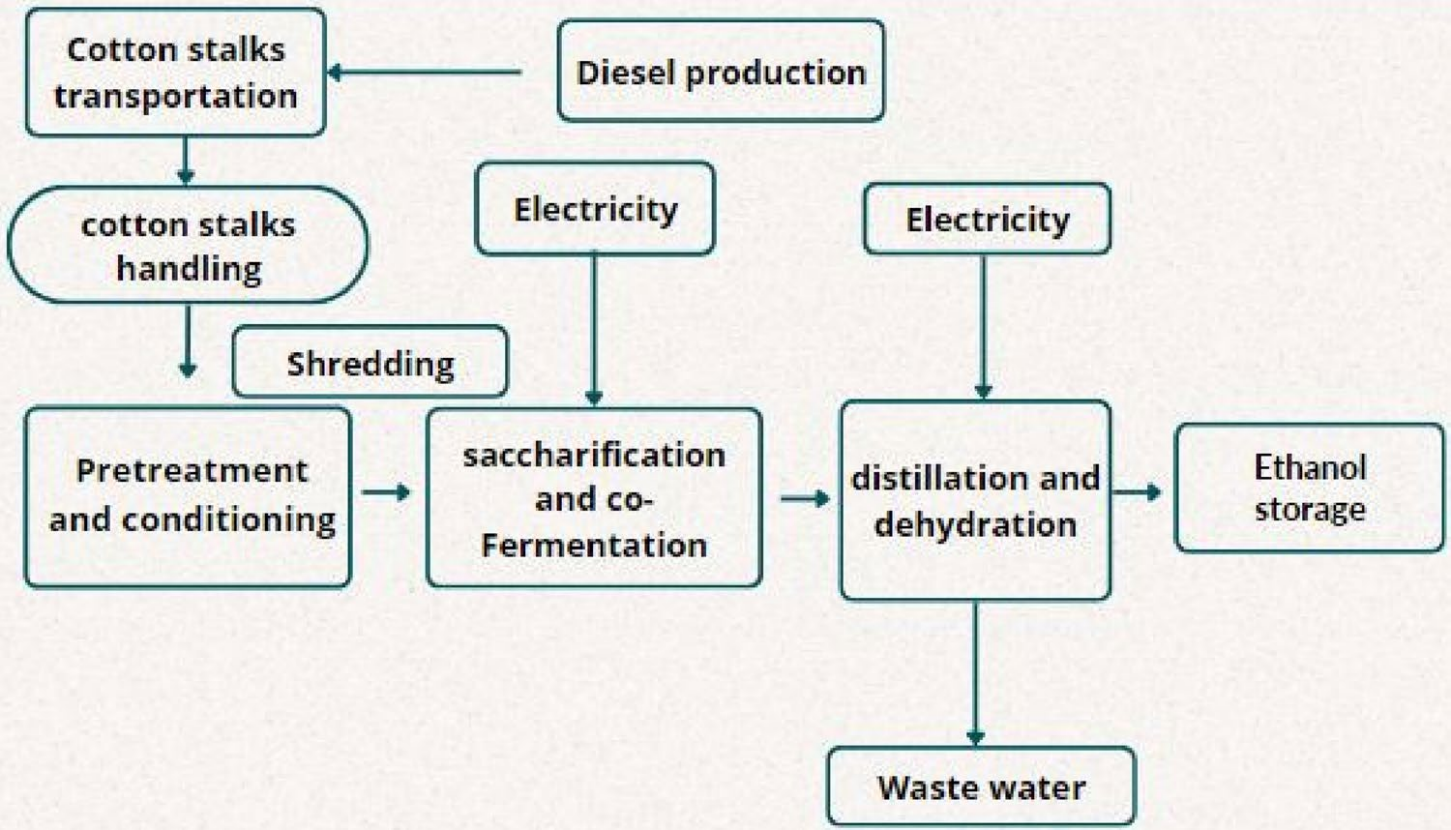
System boundaries for cotton stalks-based bioethanol.

The ethanol production process starts with four major stages firstly, preprocessing, pre-treatment, hydrolysis and fermentation, and separation, Bioethanol produced from cotton stalk waste is also a more environmentally friendly fuel than coal due to decreased carbon and ash content, Cotton stalks have high cellulose (32–46%) and hemicellulose (20–28%) content^27^. As a result, it could be utilized as a fuel for power plants as well as a source of bio-energy^37^. Cotton waste had the potential to be used for composting, briquetting, biochar, bio-oil, and the production of bioethanol^37^. As shown in Fig. 1. Pre-processing is a size-decreasing step, which was reduced using a hammer mill. The hammer mill needed more electricity as its size decreased. This study did not assume any loss of biomass during the pre-processing step. The assumed key parameters for this process were cotton stalk cultivation, transportation, chipping with a woodchipper, and ethanol fermentation process selected from the Ecoinvent 3.6 database. Pretreatment made the waste product more accessible to cellulolytic enzymes, enabling the efficient transformation of cellulose to glucose after the process of enzymatic hydrolysis^37^. The pretreatment technique considered was an alkaline pretreatment process. Ethanol recovery and separation steps were required to produce the final ethanol product. The evaluation of ethanol production from the selected feedstock involved a cradle-to-gate analysis, which considered all the stages from cultivation of the feedstock, transportation to the mill, and processing to ethanol. Additionally, the analysis included end-of-life emissions to facilitate a comprehensive comparison of greenhouse gas (GHG) emissions associated with bio-based and fossil-based ethanol. The selected inputs of the bioethanol production process were (1) cotton stalks as biowaste, (2) transportation to the storage facility, (3) Chipping with a wood chipper, (4) Finally, cotton stalks were transported to a biorefinery facility for biochemical conversion to ethanol, biochemical conversion process including all the process parameters such as fermentation activator, alkaline pretreatment, filtration, distillation. The assumptions such as cotton stalks production were considered as the feedstock, and the inventory data for cotton stalks production was taken from the Ecoinvent 3.6 database. Each light commercial vehicle was supposed to transport the waste 1000 km distance^27^. The energy requirement for the bioethanol production process was reported by Ref.^32^ equals 2.27–3.55 MJ/L EtOH. While 246.67 L and 52.5 MJ/L are the assumed ethanol yield and the thermal energy cost as output data respectively^32^. Wastewater produced from the ethanol production process ranged between 232 to 672 L/L of bioethanol^35^. The functional unit of the ethanol production process is L/kg. The system boundaries for the bioethanol production process are shown in Fig. 2.

#### Inventory of bioethanol production from cotton stalks

Assumptions of the bioethanol production process were (1) transportation of cotton stalks from farm land to the bioethanol plant for a distance of 1000 km by diesel lorry. (2) The pretreatment settings and following enzymatic saccharification findings were drawn from the scientific literature^38^. (3) The factory has processed 1000 dry metric tonnes of cotton stalks. (4) Cotton waste was transported in bulk to the bioethanol plant and was then chipped, and transferred to where alkaline pretreatment is undertaken. (4) The treated cotton stalks were separated into two areas: hydrolysis and fermentation where polysaccharides were hydrolyzed to monomer sugars, which were both fermented to ethanol by the yeast saccharomyces cerevisiae yeast. Enzymatic saccharification and hydrolysation processes were carried out for 5 days^38^.

#### Biogas production scenario

**Figure 3:**
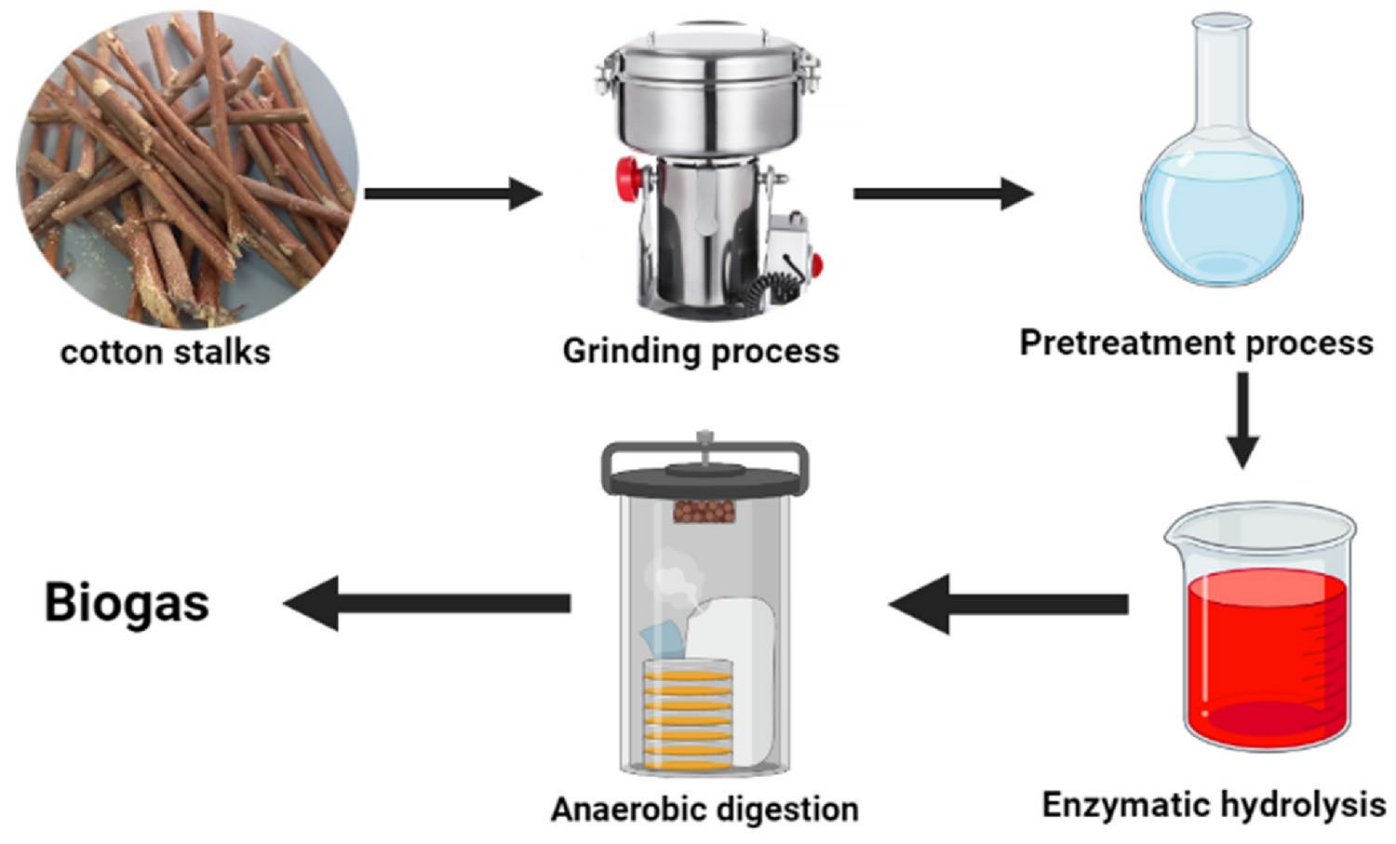
Diagram of biogas production process flow showing the steps for lignocellulosic biogas production.

A biological, economical, and environmentally friendly method for recycling biomass such as anaerobic digestion is shown in Fig. 3. The composition of the biogas produced usually consists of 50–75% CH_4_, 30–50% CO_2_, 0–3% N_2_, 6% H_2_O, and 0–1% O_2_. Other minor contaminants like hydrogen sulfide, hydrogen, ammonia, nitrogen, and water vapors could have also been present in biogas. Typically, an anaerobic digestion process consists of four sequential steps: hydrolysis, acidogenic, acetogenesis, and methanogenesis. Catalyzed by various microorganisms. Carbohydrates in cotton stalks, such as cellulose and hemicelluloses, could be transformed into a range of useful forms of energy, including bioethanol, biohydrogen, and biogas. Biogas is receiving more interest among all biofuels since its production process and conditions are easier than those of other biofuels, making the production of biogas more practical. The cradle-to-gate assessment technique of cotton stalks is critical for evaluating the impact of bioproduction processes. The life cycle study of cotton stalk biogas entails assessing numerous environmental factors that affect the balance of the natural ecosystem or environment, as well as quantifying the ecological benefits of replacing the conventional system. Furthermore. LCA may serve as a tool for both customers and regulators in determining the most environmentally friendly fuel. Anaerobic digestion (AD) is a traditional biowaste conversion method that employs microorganisms to transform organic matter into biogas and bioproducts. Anaerobic digestion is a more inexpensive way of conversion than other methods^39^. Anaerobic digestion (AD) is a three-stage process that includes hydrolysis and methanogenesis^40^. Whereas hydrolysis was the initial stage of anaerobic digestion at which complex biopolymers were converted into simple soluble compounds^41^. The critical parameters for the biogas production process were harvesting the biomass, transportation, chipping with woodchipper, biomass alkaline pretreatment, and electricity for the used reactor. The process of anaerobic digestion for biogas production was selected from the EcoInvent 3.6 database. Energy consumption in the biogas production process is 186 KWel per ton of cotton stalks^33^. The biogas yield was estimated to be 192 ml methane/g cotton stalks^36^. The functional unit of the biogas production process is L/kg.

**Figure 4:**
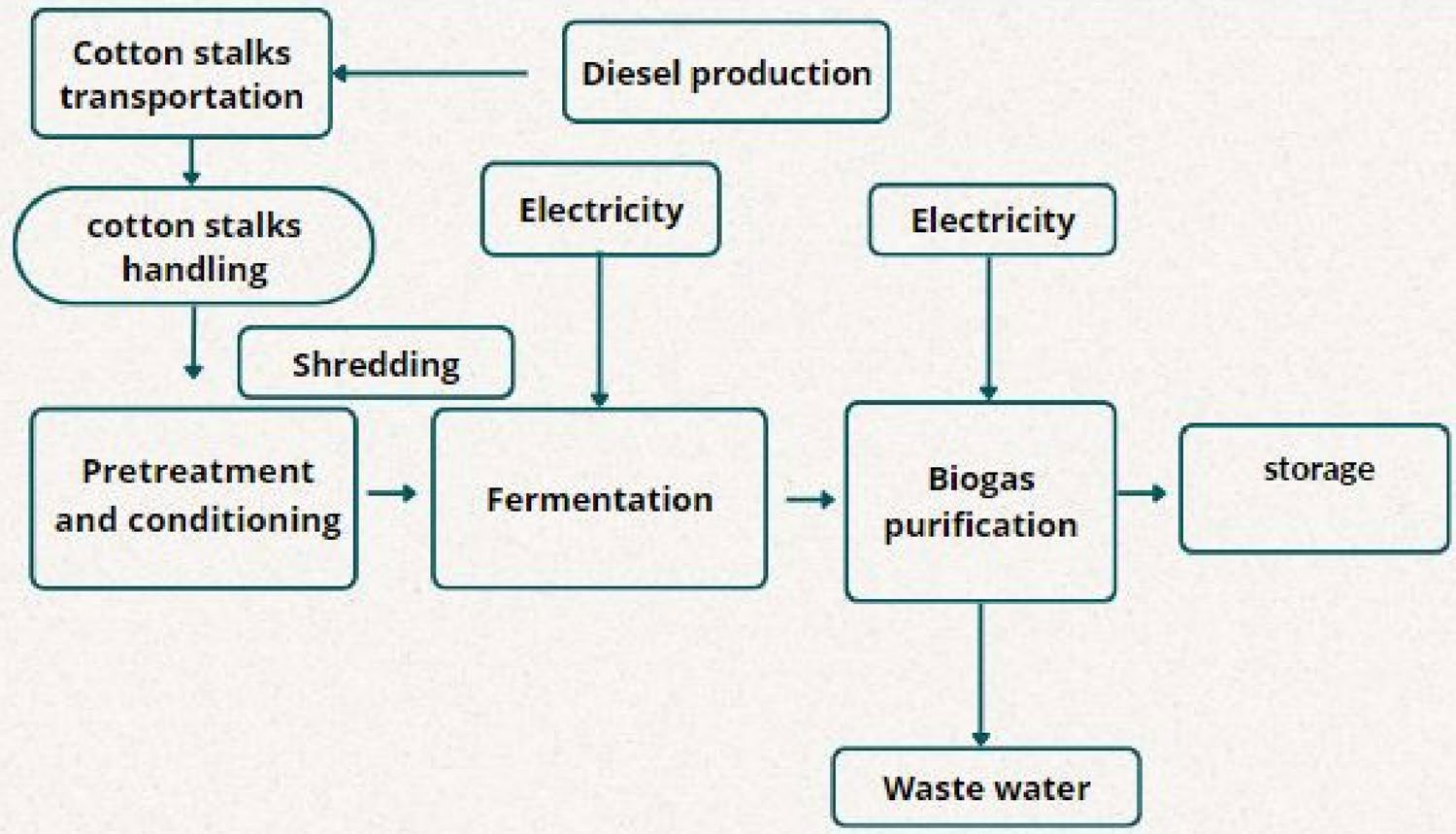
System boundaries of the biogas production process.

Cotton stalks-based composite. The system boundaries for the biogas production scenario are shown in Fig. 4.

#### Inventory data of biogas production from cotton stalks

The assumptions of the biogas production process were the transportation of cotton stalks from farmland to the Biogas production plant for a distance of 1000 km by diesel lorry. The pretreatment settings and following enzymatic saccharification findings were drawn from the scientific literature^42^. The factory processed 1000 dry metric tonnes of cotton stalks. Cotton waste was transported in bulk to the biogas production plant and was then chipped, and transferred to where alkaline pretreatment is undertaken. The treated cotton stalks were inoculated for anaerobic digestion.

### Ethical approval

The collection and handling of agricultural waste was by all the relevant guidelines.

## Cotton stalks-based wood composite manufacturing scenario

**Figure 5:**
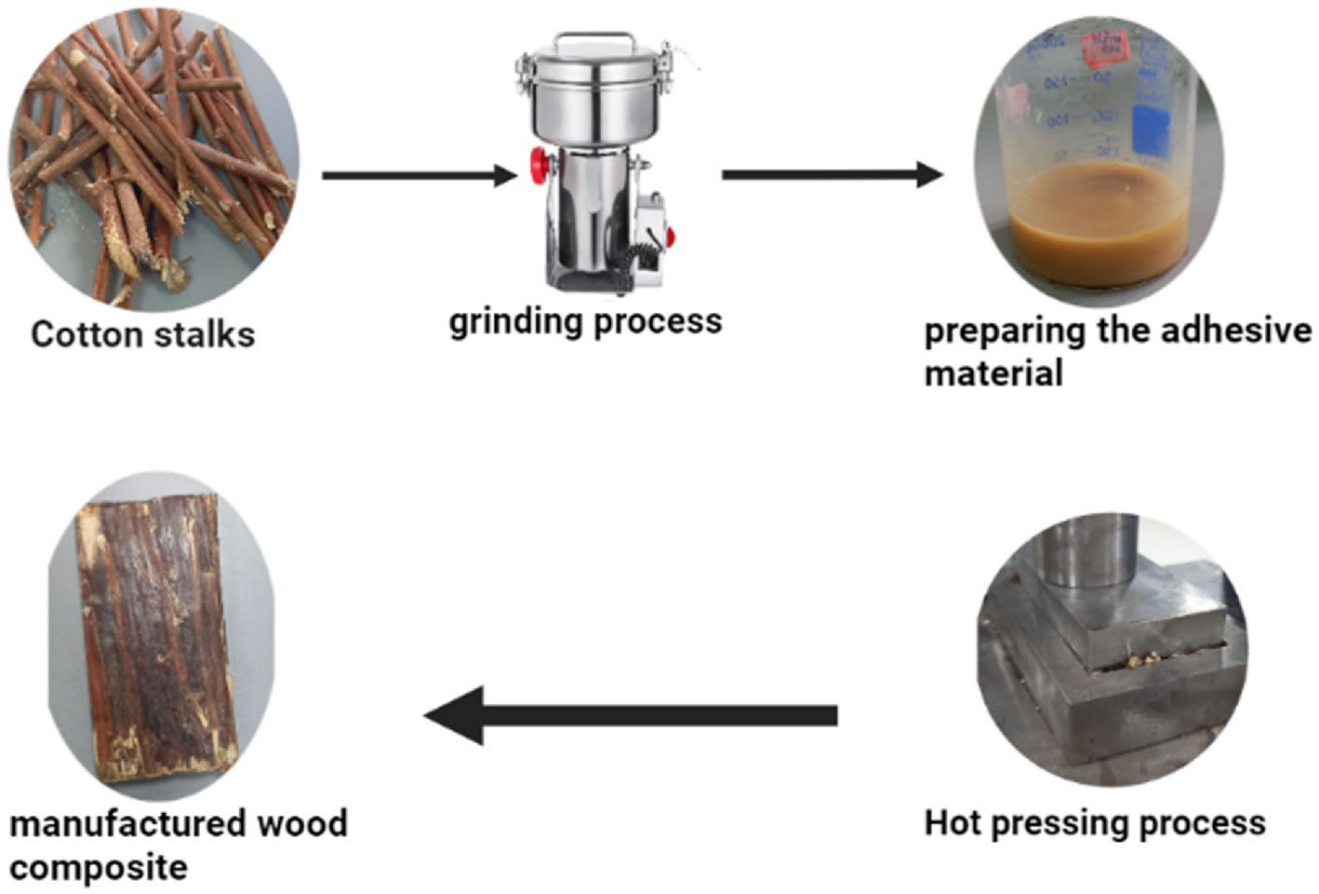
Diagram of cotton stalks-based wood composite production process flow showing the steps for cotton waste utilization.

**Figure 6:**
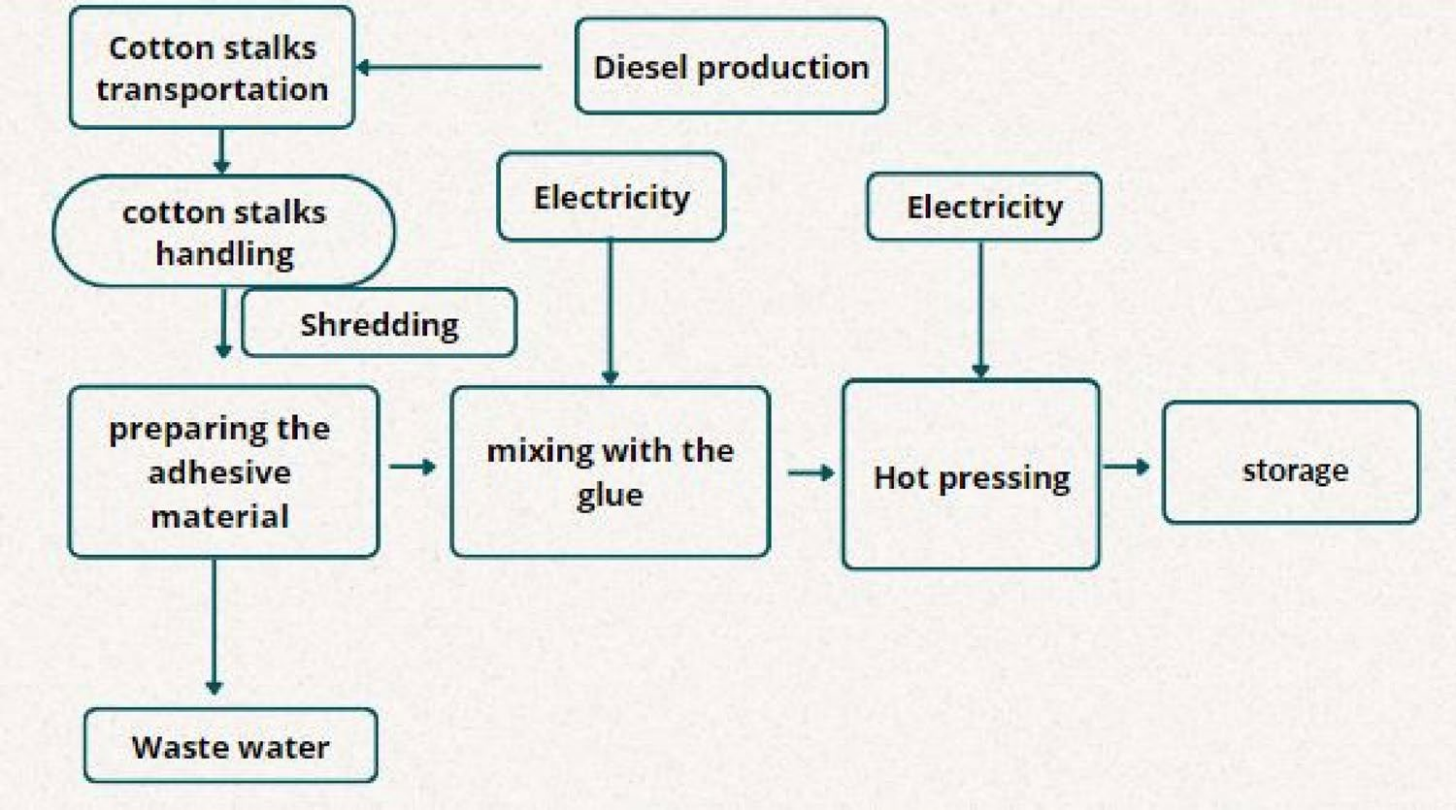
System boundaries of the cotton stalks-based composite production.

Cotton stalks and ground cotton stalks were used as raw materials for the manufacturing of wood composites utilizing soy protein and urea formaldehyde as an adhesive material. Harvested cotton stalks were pressed with the ground cotton stalks with the addition of soy protein adhesive combined with urea–formaldehyde resin. The key parameters included in the life cycle assessment process started with the transportation of 1 ton of waste material. (1) Cotton stalks were ground using a woodchipper, (2) the adhesive material using soybean, water, and urea–formaldehyde resin, and (3) the hot-pressing process was the third step using a hydraulic press machine^10^. The inputs used were based on experimental work done at Nile University, Egypt. The steps of the experimental work were illustrated in Fig. 5. The selected inputs were (1) cotton stalks as biowaste, (2) transportation to the storage facility, (3) chipping with a wood chipper, (3) preparation of adhesive material and (4) finally, cotton stalks were transported to a facility for hot pressing process. The volume of the manufactured board is the functional unit of the LCA (Figs. 4, 5). The system boundaries for the cotton stalks-based wood composite are shown in Fig. 6.

### Inventory of cotton stalks-based composite

The cotton stalks-based wood composite was manufactured at Nile University, Egypt. 1000 kg cotton stalks were transported to the storage facility using a lorry 1000 km, then it was transported to the production facility to be chopped, 25% of the adhesive material which is soybean 20% (w/w) combined with 5% (w/w) urea formaldehyde resin was prepared and then the composite was prepared using a hot-pressing electrical machine.

## Sensitivity analysis

The LCA analysis comprises multiple unclear factors, so a sensitivity analysis has been built to determine the impact of alterations in the most significant of these parameters. The methodology used in the analysis of these alternative scenarios follows the same criteria used in the LCA made in the base case, maintaining the same assumption and limitations, the same allocations and impact categories, and the same criteria in the data quality established previously.

## Uncertainty of input secondary data

Secondary data, which was collected from referenced literature, were typically related to resources and emissions associated with a given process, with a particular technology, and with a particular piece of manufacturing equipment. In the best circumstances metadata was added to secondary data to offer qualitative insights, such as limits of the system and allocation guidelines, to determine whether such data may have described the examined system. Using secondary data considerably increased uncertainty in LCA research. This is mostly because their accuracy and dependability, as well as their gathering method, were unknown^43^ Specifically, the investigation of the potential outcomes that arise from considering the substitution of 1000 kg with a longer distance of 2000 kg of cotton stalks waste in three scenarios (1) bioethanol production, (2) biogas production, and (3) wood composite manufacturing. Sensitivity analysis has been conducted. This analysis would explore the impact of increasing the quantity of cotton stalk waste used in the analysis and could investigate potential outcomes from substituting a larger quantity of cotton stalk waste.

### Transportation

Transportation secondary data was obtained from the EcoInvent database to assess the environmental implications of cotton stalks waste transportation roads. Some of the databases, for example, include steps such as infrastructure construction such as bridges tunnels, and roads and truck production, direct energy consumption and emissions during operation, end-of-life, and waste generation, whereas other databases only provide data on direct emissions by truck such as the EcoInvent database^43^. A scenario analysis was carried out using the Impact 2002 + assessment method, to compare the effect of a longer transportation distance (2000km) of cotton stalk waste for the three cotton stalk application scenarios, scenario 1: bioethanol production, (2) biogas production, (3) wood composite production.

## Limitations

While this study provided a useful comparison of various waste utilization options for cotton stalks, some limitations should have been noted. The life cycle assessment solely addressed cradle-to-gate impacts, ignoring consequences beyond the factory gate, such as during product use and end-of-life. A thorough life cycle analysis could provide further information. Just three utilization strategies were evaluated, reflecting only a portion of possible alternatives. Additional tactics may have needed to be considered to determine the best approach. The study relied on a collection of impact assessment approaches, each with its own set of limitations. Using extra or alternative impact assessment approaches may have resulted in different outcomes. Finally, the research was restricted to a specific geographical location. Because farming practices and energy infrastructures differ by region, the findings may not be applicable to other cotton-producing areas. Including more regions in the evaluation could increase generalization. Overall, the study had a limited scope in terms of life cycle boundaries, alternatives investigated, and the effect of assessment methodologies. Additional studies could be conducted to expand on these findings while resolving the constraints.

## Results and discussion

The results of the LCIA are illustrated in Tables 2, 3 and 4. This study attempts to find the main contributors to environmental impacts such as ecological footprint, human toxicity, terrestrial ecotoxicity, climate change, and CO_2_ emissions, A total number of 12 environmental assessments were examined using the open-LCA software tool. Morales et al.^44^ conducted a life cycle assessment of bioethanol production from lignocellulosic residues to compare its environmental impacts with conventional fossil fuel. It was concluded that Lignocellulosic residues such as the ones used in bioethanol production instead of fossil fuels have decreased global warming potential, abiotic depletion, and ozone layer depletion impact categories, but their effects on acidification and eutrophication were more excellent because they emitted more nitrogen oxides and sulfur oxides and other greenhouse gases. Tonini et al.^19^ conducted a life cycle assessment and provided a greenhouse gas emission for bioelectricity, biomethane, and bioethanol produced from annual crops and residues. It was concluded that greenhouse gas emissions from bioethanol production utilizing sugar beet were between 20 and 49 g CO_2_ eq, and between 20 and 90 g CO_2_ from wheat grain. According to a study conducted by Zhang, et al.^45^, a study that evaluated fossil energy and carbon footprints of soybean production in China so that key environmental impact can be recognized. The global warming and the terrestrial ecotoxicity of the production process of 1 kg of soybean adhesive were 2.79 kg CO_2_ and 6.36 kg 1,4-DCB 6.36, respectively, this explains that the terrestrial ecotoxicity of the wood composite production scenario (2.538 Kg1,4-DCB-eq), was higher than that of bioethanol (0.734 Kg1,4-DCB-eq) and biogas production scenarios (0.768 Kg1,4-DCB-eq). Limitations of the lifecycle assessment in this work might be the data Availability and quality. Inadequate and out-of-date data might lead to uncertainty and restrictions in the outcomes. Furthermore, data correctness and representativeness can vary, introducing biases or inaccuracies in the assessment. Variations in system boundaries and functional units can make direct comparisons of various production scenarios difficult.

**Table 2:** Results of LCA of bioethanol production from cotton stalks waste.

Impact assessment method	Unit	Impact category	Impact result
Ecological footprint	M^2^a	Total-CO_2_	210.3158
Greenhouse gas protocol	KgCO_2_ eq	Biogenic CO_2_	375.817
Greenhouse gas protocol	KgCO_2_	Fossil CO_2_	126.477
IMPACT 2002 +	Points	Climate change-total	0.011300
IMPACT 2002 +	Points	Human health-total	0.01239
Ecosystem damage potential	Points	Land occupation	190.96915
Ecosystem damage potential	Points	Land use	274.01535
CML-IA baseline	KgSO_2_	Acidification	4.840
IMPACTWORLD +	DALY	Human toxicity cancer, long term	8.11022E − 7
IMPACT World +	DALY	Ozone layer depletion	1.517
Recipe midpoint	kgCO_2_-eq	Climate change-GWP20	190.28455
Recipe midpoint	Kg1,4-DCB-eq	Terrestrial ecotoxicity	0.73498
Cumulative energy demand	MJ-eq	Solar-renewable energy resources	0.65821
Recipe midpoint	Kg1,4-DCB Eq	Human toxicity-HTP100	51.5763

**Table 3:** Lifecycle impact results of biogas production from cotton stalks waste.

Impact assessment method	Unit	Impact category	Impact result
Ecological footprint	M^2^a	Total-total	224.260
Ecological footprint	M^2^a	Total CO_2_	194.727
Greenhouse gas protocol	KgCO_2_ eq	Fossil CO_2_	94.29
Greenhouse gas protocol	KgCO_2_	Biogenic CO_2_	652.64
Impact 2002 +	Points	Climate change-total	0.01083
Impact 2002 +	Points	Human health-total	0.01569
Ecosystem damage potential	Points	Land occupation	4.593
Ecosystem damage potential	Points	Land use	4.7713
CML-IA baseline	KgSO_2_	Acidification	0.777
CML-IA baseline	Kg 1,4-DB eq	Human toxicity	35.017
Impact world	DALY	Ozone layer depletion	3.416
Recipe midpoint	Kg 1,4-DCB eq	Terrestrial ecotoxicity	0.76873
Recipe midpoint	KgCO_2_ eq	Climate change GWP	109.997

**Table 4:** LCA of cotton stalks waste wood composites production process.

Impact assessment method	Unit	Impact category	Impact result
Ecological footprint	M^2^a	Total footprint	2144.431
Greenhouse gas protocol	KgCO_2_ eq	Fossil CO_2_ eq	206.95
Greenhouse gas protocol	KgCO_2_	Biogenic CO_2_ eq	377.51
Recipe midpoint	KgCO_2_ eq	Climate change GWP20	279.57
Impact world	DALY	Ozone layer depletion	4.1160
Impact 2002 +	Points	Climate change-total	0.01761
Impact 2002 +	Points	Human health-total	0.05299
Impact 2002 +	Points	Ecosystem quality-total	0.07419
Recipe midpoint	Kg 1,4-DCB-eq	Terrestrial ecotoxicity	2.5389
Ecosystem damage potential	Points	Land use	0.36132
Ecosystem damage potential	Points	Land occupation	510.26
CML IA baseline	Kg 1,4 DB eq	Human toxicity	31
CML IA baseline	Kg SO_2_ eq	Acidification	1.712

As shown in Table 2 impact results of bioethanol production were evaluated using several impact categories human toxicity, terrestrial ecotoxicity, climate change, total, biogenic, and fossil CO_2_, land occupation, and use. The biogas production scenario has a 109.99 kgCO_2_ climate change impact, which is lower than the climate change impact results of both bioethanol and wood composite production which are 190.28 kgCO_2_ and 279.57 kgCO_2_ respectively. The ecological footprint impact of the bioethanol synthesis scenario has the lowest value of 210.315 kgCO_2_, then biogas (224.26 kgCO_2_) and wood composite production processes (2144.4 kgCO_2_). Xu et al.^46^, have conducted a lifecycle assessment for biogas production scenarios, and the results showed that the impacts of human toxicity had a major impact on the overall ecological impact. Wood composite production has a lower human toxicity impact of (31 kg1,4-DCB eq) than biogas production scenario (35.017 kg1,4-DCB eq) and bioethanol production scenario (51.57 kg1,4-DCB eq). There are some potential explanations for human toxicity and ecotoxicity that should be considered when comparing the environmental implications of bioethanol production to other scenarios. Workers' exposure to occupational hazards such as harmful air pollutants and chemicals during bioethanol production scenarios may have a negative impact on human health. Chemicals, like enzymes or acids, are used in the fermentation and distillation processes to convert feedstocks into ethanol. The handling and storage of these compounds can endanger human health if not carefully handled. The ozone layer depletion impact result of the bioethanol production scenario equals 1.517 DALY which is lower than both wood composite (4.1160 DALY) and biogas production scenarios (3.416 DALY). The biogenic CO_2_ category result shows that the biogas production process (652.64 KgCO_2_) had a higher impact than both the bioethanol (375.817) and wood composite production scenarios (377.51 kgCO_2_) , in contrast with the fossil CO_2_ impact result which is lower than both the bioethanol and the wood composite production. It was reported that the greenhouse gas emission of utilizing 1 ton of rice straw waste to produce bioethanol was 288 kg fossil CO_2_ equation^18^. Regarding fossil CO_2_ the manufacturing of cotton stalks-based wood composite has an ecological impact higher than that of bioethanol and biogas production processes. This means that the wood composite production scenario consumed more fossil resources than the other 2 scenarios, followed by the bioethanol production scenario. Acidification as an emission was clearly higher in the bioethanol production scenario (4.840 kgSO_2_) compared with the biogas (0.777 kgSO_2_) and cotton stalks-based wood composite production scenario (1.712 kgSO_2_). In summary, biogas production generally exhibits lower climate change impact and lower carbon footprint compared to both bioethanol and wood composite production. Bioethanol production has the lowest carbon footprint but a higher human toxicity impact. Bioethanol also has a lower ozone layer depletion impact compared to wood composite and biogas production. Wood composite production has a higher ecological impact and consumes more fossil resources compared to bioethanol and biogas production. Acidification impact is highest in the bioethanol production scenario. Sensitivity analysis results after doubling transportation distance and the energy consumption are shown in Tables 5 and 6 respectively.

**Table 5:** Sensitivity results after doubling the transportation distance.

Doubling the transportation distance		
Scenario 1	Scenario 2	Scenario 3
Climate change impact: 0.0212 points	Climate change impact: 0.01098 points	Climate change impact: 0.0188 points
Human health impact: 0.0231 points	Human health impact: 0.01582 points	Human health impact: 0.05582 points

**Table 6:** Sensitivity results after doubling the energy consumption.

Doubling the energy consumption		
Scenario 1	Scenario 2	Scenario 3
Climate change impact: 0.0205	Climate change impact: 0.01563	Climate change impact: 0.01808
Human health impact: 0.02654	Human health impact: 0.02986	Human health impact: 0.05637

### Effect of doubling transportation distance on climate change's impact

- Scenario 1: increased by 0.99%
- Scenario 2: increased by 0.015%
- Scenario 3: increased by 0.119%.

### Effect of doubling transportation distance on human health impact

- Scenario 1: increased by 1.071%
- Scenario2: increased by 0.013%
- Scenario 3: increased by 0.292%.

### Effect of doubling energy consumption on climate change impact

- Scenario 1: increased by 0.92%
- Scenario 2: increased by 0.48%
- Scenario 3: increased by 0.047%.

### Effect of doubling energy consumption on human health impact

- Scenario 1: increased by 1.415%
- Scenario 2: increased by 1.417%
- Scenario3: increased by 0.338%.

Sensitivity analysis results deduced that considering climate change impact, the bioethanol production scenario has the highest sensitivity, it was observed that larger numbers of transportation distances or the energy consumed in each scenario marked an increase on both climate change and human health impact of the production process, from the date of the sensitivity analysis, a remarkable sensitivity appeared in the bioethanol production scenario regarding climate change impact category. Also, regarding human health impact, bioethanol production showed a remarkable increase that other application scenarios. After the bioethanol production scenario, the wood composite production scenario had an increase in both climate change and human health impact categories. These results deduced that doubling the transportation distance has led to a moderate elevation in environmental loads^47^. The data presented illustrates how doubling energy usage and transportation distance might affect human health and the impact of climate change in the three scenarios. According to the results, the impact on human health and climate change increases when transportation distance doubles. When the transportation distance is doubled, Scenario 1 shows the greatest rise in the impact on human health (1.071%) and the impact on climate change (0.99%). This indicates that larger travel distances had a negative impact on human health due to variables including increased air pollution and exposure to risks associated with transportation, as well as a large contribution to greenhouse gas emissions. Doubling energy use also had a greater impact on human health and climate change. When energy consumption was doubled, Scenario 1 exhibited the largest rise in the impact on human health (1.415%) and the impact on climate change (0.92%). This suggested that increasing energy consumption could have a negative impact on human health due to pollution from the processes involved in the production and consumption of energy, as well as increased emissions of greenhouse gases that fuel climate change. When comparing the scenarios, we find that Scenario 2 constantly showed the least amount of rise in the impact on human health and the impact on climate change regarding energy consumption and transportation distance. This indicated that, even with the doubled energy or transportation distance, Scenario 2 which is the biogas production process had less sensitivity than both Scenario 1 and 3. The information emphasizes the significance of sustainable energy and transportation practices overall. It highlights the necessity of reducing travel lengths by implementing effective logistics systems and embracing alternate forms of transportation. Furthermore, the adverse effects on climate change and human health can be considerably reduced by increasing energy efficiency, using renewable energy resources, and lowering energy use.

## Conclusion

In this article, a detailed assessment of the environmental impacts of three different applications from cradle to gate was conducted using open LCA software. Life cycle impact assessment of wood composite, bioethanol, and biogas production utilizing 1000 kg cotton stalks were evaluated. 12 different impact categories were imported in this study. Climate change GWP20, ozone layer depletion, terrestrial ecotoxicity, human toxicity land use and occupation, and acidification are examples of impact categories that were assessed in this study. Using impact categories such as climate change, terrestrial ecotoxicity, human toxicity, and ozone layer depletion potential in LCA is important as it allows for a comprehensive assessment of environmental and health impacts, facilitates identification of hotspots and trade-offs, supports policy compliance, and addresses stakeholder concerns. Transportation and landfilling stages were not included in the cradle-to-gate lifecycle assessment. It corresponded that ethanol produced from lignocellulosic materials reduces the depletion of greenhouse gases and the consumption of fossil fuels. The total climate change of wood composite, bioethanol, and biogas production processes was 0.01761, 0.011300, and 0.01083 points respectively. The terrestrial ecotoxicity of the wood composite production scenario (2.538 Kg1,4-DCB-eq), was higher than that of bioethanol (0.734 Kg1,4-DCB-eq) and biogas production scenarios (0.768 Kg1,4-DCB-eq). The human toxicity impact result of the wood composite production, biogas, and bioethanol production scenarios is 31 kg 1,4 DB eq, 35.017 kg 1,4 DB eq, 51.5763 kg 1,4 DB eq respectively. The ozone layer depletion impact results of the biogas production scenario, wood composite, and bioethanol production scenarios are 3.416, 4.1160, and 1.517 DALY. It was concluded that utilizing 1 ton of cotton stalk waste in wood composite production has a lower CO_2_ impact than its utilization in bioethanol and biogas production processes. While biogas production process has a lower contribution to climate change compared to wood composite and bioethanol production processes. Regarding human toxicity and ozone layer depletion bioethanol production has a lower environmental impact. The LCA results can be used to identify the opportunities for reducing the environmental impact of each application. While these findings offer significant insights, it is advisable to consider other factors such as economic viability, technological advancement, societal acceptance, and governmental backing to attain a more all-encompassing comprehension of the possibilities for cotton stalk utilization. This research emphasizes the significance of adopting a systems approach in the management of agricultural waste. It brings to light the potential of cotton stalk, a prevalent agricultural waste byproduct, to be utilized in various industries such as construction, energy, and agriculture to improve environmental sustainability. The results of a life cycle assessment (LCA) provide valuable insights into reducing the negative environmental effects of every application by identifying the key stages, processes, and materials with the highest ecological burdens, allowing for targeted enhancements, informed choices, and the establishment of more sustainable strategies and practices throughout the entire life cycle. Additional investigation is required to enhance the methodology and encompass alternative potential uses of cotton stalks. Through this approach, it is possible to further enhance the utilization of this agricultural residue to promote a sustainable and resilient future.

## Data Availability

All data generated or analyzed during this study are included in this published article.

## Abbreviations

LCA: Lifecycle assessment
LCIA: Lifecycle impact assessment
LCI: Lifecycle inventory
GHG: Greenhouse gas
GWP: Global warming potential
CED: Cumulative energy demand
AD: Anaerobic digestion
CML: Centre of environmental science
GMP: Greenhouse gas management potential
